# Detection of *Bartonella henselae* in domestic cats' saliva

**Published:** 2010-06

**Authors:** K Oskouizadeh, T Zahraei-Salehi, SJ Aledavood

**Affiliations:** 1 Department of Clinical Sciences, Faculty of Veterinary Medicine, Shahid Chamran University of Ahvaz, Ahvaz, Iran; 2 Departments of Microbiology; 3 Clinical Sciences, Faculty of Veterinary Medicine, University of Tehran, Tehran, Iran

**Keywords:** *Bartonella henselae*, cat, cat scratch disease, Iran

## Abstract

**Background and Objectives:**

*Bartonella* species are being recognized as increasingly important bacterial pathogens in veterinary and human medicine. These organisms can be transmitted by an arthropod vector or alternatively by animal scratches or bites. The objectives of this study were to identify contamination of cat's saliva and nail with *B. henselae* as a causative role to infect human in a sample of the cat population in Iran.

**Materials and Methods:**

Blood, saliva and nail samples were collected from 140 domestic cats (stray and pet) from Tehran and Shahrekord and analyzed for the presence of *B. henselae* with cultural and polymerase chain reaction (PCR) methods and DNA sequencing.

**Results:**

In this study *B. henselae* was detected in 10.9% of saliva samples (12/110) from pet cats*. B. henselae* was not detected in nail samples of pet cats (n=110), and in any feral cats’ saliva and nail samples (n=30).

**Conclusion:**

Our data suggest that pet cats are more likely than stray cats to infect human with *B. henselae* after a bite and also stray cats can play a role as a reservoir for this bacteria. This is the first report that investigates the presence of *B. henselae* in cats oral cavity in Iran.

## INTRODUCTION

*Bartonella* species are being recognized as increasingly important bacterial pathogens in veterinary and human medicine. These organisms can be transmitted by an arthropod vector or alternatively by animal scratches or bites (1). Among the 11 species or subspecies known or suspected to be pathogenic in humans, 8 have been detected in or isolated from pet dogs or cats, thereby highlighting the zoonotic potential of these bacteria (2). Although cat scratch disease (CSD) was recognized in 1930 and first reported by Debrè *et al*., the etiological agent was identified only in 1992 when *Bartonella henselae* was definitely associated with CSD in humans (3–5). *Bartonella henselae*, the etiologic agent of CSD, has been identified as a cause of bacillary angiomatosis in immunocompromised persons (6, 7). Pet ownership is an extremely common phenomenon worldwide, with a tradition that dates back 15,000 years or more. Pet- associated zoonoses can include skin and soft tissue syndromes secondary to bites, scratches, and other direct contact, septicemia from contamination of intravascular and other indwelling medical hardware, parasitic syndromes, gastroenteritis, viral pathogens, and other zoonoses. An emerging pathogen among dogs and cats is *Bartonella henselae.* CSD is quite common and affected approximately 25000 people annually in the United States (8). The objectives of this study were to identify contamination of cats’ saliva and nail with *B. henselae* as a causative role to infect human in a sample of the cat population of Tehran and Shahrekord, Iran.

## MATERIALS AND METHODS

Blood, saliva and nail samples were collected from healthy pet cats (n=110) and stray cats (n=30) at the Veterinary Medical Teaching Clinic of Tehran and Shahrekord University, Iran. All samples were collected from June 2005 to November 2007. Stray cats were caught by automatic box trap. All cats were examined physically, and healthy cats without any infectious diseases or antibiotic therapies during one month before of the study, were selected. None of these cats were infested with fleas at the time of sampling.

**Isolation of *B. henselae* from cat's blood (culture method).** EDTA tubes were filled with 2 ml of blood from the external jugular vein of each cat under aseptic condition and they were stored at −70°C for 3 to 4 weeks. After this time, samples were defrosted, tubes were centrifuged at 3000×g for 30 min at room temperature and the pellets were inoculated onto a 5% fresh sheep blood agar plates. The Brucella growth supplement (Mast, Merseyside, UK) containing polymixin B, bacitracin, natamycin, nalidixic acid, nystatin, and vancomycin was added to the plates to inhibit the growth of other micro organisms. The plates were then incubated at 35°C under conditions of 5–7% CO_2_ and humidity of>40% for 4 to 8 weeks (9, 10).

**Isolation of *B. henselae* from cat's mouth and paw (culture method).** An oral swab was collected using a sterile cotton applicator. The swab was placed against the inside surface of the cat's cheek. Saliva was collected by rolling the swab against the cheek and to satisfy sampling from the paw; swab was tainted with nutrient broth. Specimen from the paw was cultured directly on chocolate agar plate. Subsequently, the oral swabs were suspended in 1ml of nutrient broth, and the broth was diluted by a factor up to 10^−9^. From each dilution, 0.1 ml was inoculated onto chocolate agar plate and the plates were incubated as described above.

After gram staining and microscopic examination of the colonies, differential characteristics of commonly pathogenic *Bartonella* spp. were investigated using standard methods. Finally, the strains were subjected to PCR to identify the species of the isolated organisms (9).

**DNA extraction for PCR amplification.** The strains were grown on chocolate or blood agar plates. Total genomic DNA was extracted from samples using the commercial kit (Qiagen, Hilden, Germany, genomic DNA purification kit). The procedures provided by the manufacturer were followed. The extracted DNA was used as a template in the PCR assay. Purified DNA from *B. henselae* ATCC 49793 provided by Giladi (11) was used as a positive control in PCR experiments.

**PCR Assay and sequencing.** Primers CAT1 (5′- GATTCAATTGGTTTGAAGGAGGCT-3′) and CAT_2_ (5′- TCACATCACCAGGACGTATTC-3′) were used to amplify a 414-bp fragment of *htr*A gene as described by Anderson *et al.* and Sander *et al.* (12, 13). The *htr*A DNA gene amplification was carried out in 25 µl reaction volumes containing; 5 µl of the extracted DNA sample, 2.5 µl AMS buffer 5x, 50 mM MgCl_2_, 100µM dNTP, 10 to 20 pmol of each primer (CAT_1_ and CAT_2_), 12.5 µl distilled water, and 0.5 U of *Taq* DNA polymerase (Fermentas, Vilnius, Lithuania).

The DNA amplification was performed using the following cycling conditions: initial denaturation at 95°C (5 min), followed by 30 cycles of 94°C (1min), 57°C (1min), and 72 °C (1.5 min), with a single final extension step at 72°C (7min). Standard procedures were taken to prevent contamination of the sample DNA (14).

PCR products were separated on a 1.5% agarose gel and visualized after staining with ethidium bromide. All PCR products were analyzed by sequencing with an automated sequencer ABI 3730XL Genetic Analyzer (Macrogen, Seoul, Korea).

## RESULTS

In this study *B. henselae* was detected in 10.9% of saliva samples (12/110) from pet cats. *B. henselae* was not detected in nail samples of pet cats (n=110), and in any stray cats’ saliva and nail samples (Table 1). *B. henselae* was not detected in blood samples of pet cats; in contrast it was detected in 16.6% of blood sample (5/30) from stray cats. All of the 10.9% of positive saliva samples were culture positive for *Bartonella* spp. according to morphological and biochemical characteristics.

**Table 1:** Characteristics of pet cats with *B. henselae* salivary infection.

	Cat number	Age (month)	Gender^a^	Outdoor	indoor
1	51	8	F	outdoor	
2	53	12	F	outdoor	
3	57	2	M	outdoor	
4	63	4	M	outdoor	
5	64	4	M	indoor	
6	65	4	F	indoor	
7	73	36	F	outdoor	
8	75	15	M	outdoor	
9	76	36	M	outdoor	
10	84	3	M	indoor	
11	93	18	M	outdoor	
12	94	24	M	outdoor	

a M, male; F, female

**Fig. 1:**
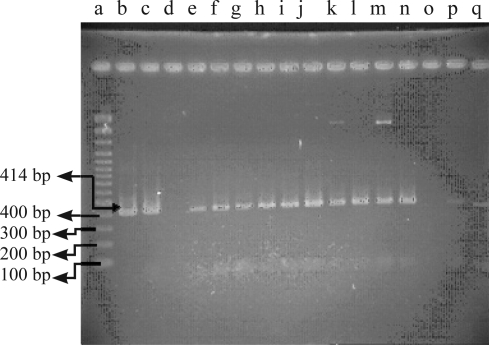
Agarose gel electrophoresis of the PCR products. a. Marker (Fermentas); b. Positive control; c. Isolate from cat 51; d. Negative control; e-n. Isolates from cats 53, 57,63,64,65, 73, 75, 76, 84, 93, 94; o,p. Negative controls; q. Blank

PCR amplification of the extracted DNA from 12 culture positive specimens for *htr*A gene produced an amplicons with 414-bp in size (Fig. 1). The *htr*A sequences obtained for *B. henselae* were submitted to the GenBank under DQ874333 and DQ874334 accession numbers.

## DISCUSSION

This is the first study that documents isolation of *B. henselae* from cats’ saliva in Iran. Up to 10.9% (12 of 110) of healthy pet cats in our study showed the presence of *B. henselae* DNA in oral swabs obtained from saliva, however *B.henselae* was not detected from the saliva of stray cats. Furthermore, our results indicated that stray cats had 16.6% (5 of 30) bacteremia with *B. henselae*. All 12 pet cats in this study lacked bacteriologic evidence of *B. henselae* infection. These data suggest that pet cats are more likely than stray cats to infect human with *B. henselae* after a bite and also stray cats can play a role as a reservoir for this bacteria.

The risk of human *Bartonella* infection from stray cats can be direct and indirect. Stray cats do not allow themselves to be stroked, and hence; their contacts with human (scratching and biting) are relatively limited and the risk of direct infection should be low. The risk of indirect infection is greater, because pet cats, which are occasionally outside, can be infected by *Bartonella* spp. either if they are scratched or bitten by stray cats (15).

Several investigators have reported a high seroprevalence and asymptomatic *B. henselae* bacteremia among naturally affected cat population (14, 16, 17). In other studies a low prevalence of bacteremia has been reported in pet cats from France (8.1%) and Japan (9.1%), whereas prevalences higher than 60% have been reported in the United States, Europe and Southeast Asia (3, 18–21). Chomel *et al.* reported the percentage of bacteremic cats harbouring *B. henselae* as 89% (17 of the 19 culture positive cats) in the Philippines with the 68% (73 of 107) of them showed 1:64≤ titers of antibodies to *B. henselae* (18). The percentage of *B. henselae* seroprevalence have been reported 23% (23 of 100) in domestic cats from Tehran, Iran (22). This study also confirmed that indoor pet cats are less frequently seropositive than outdoor pet cats or stray cats.

Koehler *et al.* found in 41% of their cats a *B. henselae* bacteremia, many of these animals had close contact to each other, and this could be a reason for the high prevalence of *B. henselae* in that study (14). However, low detection of *B. henselae* bacteremia in our study appears to be due to the fact that few of these animals had close contact to each other and no ectoparasite infestation especially flea infestation was observed.

In general, cats are implicated in the transmission of *B. henselae*, typically resulting in cat-scratch disease; however, there have also been sporadic reports of *Bartonella* transmission by dogs (17, 18, 23). *B. henselae* DNA in dogs and cats saliva was detected from the USA and Korea; however Sander *et al.* could not demonstrate *Bartonella* species from gingival swab (10, 19) (20). Recently, *Bartonella* DNA has been amplified from peripheral lymph nodes of healthy dogs (21). *B. henselae* was also amplified from salivary gland tissues from a dog with sialadenitis (22). There are several plausible routes by which a *Bartonella* spp. could gain entry to the oral cavity. Future studies should determine if the tonsillar lymphoid tissues, salivary glands, or periodontal, gingival, or other oral tissues can serve as sources of *Bartonella* spp. contamination of canine saliva. As there is no information about canine *B. henselae* infection in Iran and *Bartonella* infection may represent an occupational risk for veterinary professionals and others with extensive animal contact (23), further investigation is recommended to elucidate the role of this organism as threats to humans and animals health in Iran.
